# Comparative study of the efficacy and safety of minimally invasive interlaminar full-endoscopic discectomy versus conventional microscopic discectomy in single-level lumbar herniated intervertebral disc (ENDO-F Trial): a multicenter, prospective, randomized controlled trial protocol

**DOI:** 10.1186/s13018-022-03052-1

**Published:** 2022-03-28

**Authors:** Jin-Sung Kim, Jun Ho Lee, Junseok Bae, Dong Chan Lee, Sang-Ha Shin, Han Joong Keum, Young Soo Choi, Sang Soo Eun, Seung Ho Shin, Hyun Jin Hong, Ji Yeon Kim, Tae Hyun Kim, Woojung Lim, Junghoon Kim, Sang-Min Park, Hyun-Jin Park, Hong-Jae Lee

**Affiliations:** 1 Department of Neurosurgery, Seoul St Mary’s Hospital, College of Medicine, The Catholic University of Korea, Seoul, Republic of Korea; 2 Department of Neurosurgery, Kyung Hee University Medical Center, Seoul, Republic of Korea; 3 Department of Neurosurgery, Chungdam Wooridul Spine Hospital, Seoul, Republic of Korea; 4 Department of Neurosurgery, Wiltse Memorial Hospital, Anyang, Republic of Korea; 5 Department of Orthopaedic Surgery, Chungdam Wooridul Spine Hospital, Seoul, Republic of Korea; 6 Department of Orthopaedic Surgery, Spine Center, Seoul National University College of Medicine, Seoul National University Bundang Hospital, Seongnam, Republic of Korea; 7 Department of Orthopedic Surgery, Spine Center, Kangnam Sacred Heart Hospital, Hallym University College of Medicine, Seoul, Republic of Korea; 8 Department of Neurosurgery, Daejeon St. Mary’s Hospital, College of Medicine, The Catholic University of Korea, 64 Daeheung-ro, Jung-gu, Daejeon, 301-723 Korea

**Keywords:** Conventional microscopic discectomy, Interlaminar full-endoscopic discectomy, Lumbar disc herniation, Study protocol, Minimally invasive surgery

## Abstract

**Background:**

Advances in minimally invasive surgery have expanded the indications for interlaminar full-endoscopic discectomy. Although the clinical outcomes for this approach may be equivalent to those of conventional microscopic discectomy, the supporting evidence is still based on small, single-center, prospective, and retrospective studies. Therefore, a multicenter randomized controlled trial is warranted.

**Methods:**

This will be a prospective, multicenter, randomized controlled trial comparing the efficacy and safety of interlaminar full-endoscopic discectomy to those of conventional microscopic discectomy. The trial will enroll 100 participants with a lumbar disc herniation, 50 in each group. The primary outcome will be the Oswestry Disability Index (ODI) score at 12 months post-surgery. Secondary outcomes will be back and leg pain (visual analog scale); the ODI; the EuroQol-5-dimension score; patient satisfaction; and walking distance/time and time to return to daily activities post-surgery. Surgical outcomes will include postoperative drainage, operative time, duration of hospital stay, postoperative creatine kinase level as an indicator of muscle injury, and postoperative scarring. Postoperative magnetic resonance imaging, computed tomography, and simple radiography will be performed to evaluate radiographic outcomes between the two surgical approaches. Surgery-related complications and adverse effects will be evaluated as safety outcomes. A single assessor at each participating hospital, blinded to group allocation, will assess the enrolled participants at baseline, at 2 weeks, and at 3, 6, and 12 months postoperatively.

**Discussion:**

This trial is designed to determine whether interlaminar full-endoscopic discectomy is clinically comparable to microscopic discectomy to treat lumbar disc herniations. All efforts will be made to reduce bias, including adequate sample size, blinded analyses, and multicenter prospective registration. The outcomes will inform practice, providing the evidence needed for using interlaminar full-endoscopic over microscopic discectomy by confirming the potential of this technique to improve patient satisfaction and clinical outcomes.

*Trial registration*: Clinical Research Information Service; cris.nih.go.kr. (KCT0006277); protocol version (v1, June 8, 2021).

## Background

Discectomy is the most common surgical method for resolving lumbar radiculopathy caused by disc herniation and nerve root compression [1, 2]. Currently, microscopic discectomy is performed as minimally invasive surgery, reducing the invasiveness of conventional open discectomy [3–5]. Minimally invasive spinal surgery has been developed by using a tubular retractor, microscope, and endoscope to achieve effective neural decompression while preserving the stabilizing structures of the spine [4–7]. Although technically more demanding, interlaminar full-endoscopic discectomy has significantly reduced surgical invasiveness, thereby expanding the indications for endoscopic surgery [5, 8–10]. Specifically, interlaminar full-endoscopic discectomy offers several advantages over conventional microscopic discectomy, including a smaller skin incision and, thus, less scarring and less muscle damage, a lower infection rate and volume of blood loss, a less painful recovery, and a shorter hospital stay [3, 11–17]. Previous studies have reported no differences in clinical outcomes between interlaminar endoscopic and microscopic discectomy [18–22]. However, the evidence regarding the efficacy and safety of interlaminar full-endoscopic discectomy compared with those of microscopic discectomy is limited by the small sample size in these studies and type of research design, namely retrospective, single-center prospective designs [7, 18–25]. Therefore, a multicenter, randomized controlled trial (RCT) is warranted.

To address this gap in evidence, we propose a multicenter, prospective RCT to compare the outcomes of interlaminar full-endoscopic discectomy versus those of microscopic discectomy. Our guiding hypothesis is that the efficacy and safety of interlaminar full-endoscopic discectomy and microscopic discectomy of the lumbar spine will be similar.

In addition to the findings from previous studies, if this non-inferiority RCT with a high evidence level shows no differences in the primary outcomes of neural decompression between the two discectomy surgeries, these results, combined with less invasiveness of interlaminar full-endoscopic discectomy may suggest that interlaminar full-endoscopic discectomy is a superior alternative to conventional open surgery.

## Methods/design

### Trial design

This study aims to evaluate the non-inferiority of the outcomes of interlaminar full-endoscopic discectomy versus those of microscopic discectomy using a multicenter RCT design. Our methods have been approved by the institutional review boards of the participating hospitals (The Catholic University of Korea Seoul St Mary’s Hospital; Kyung Hee University Medical Center; Chungdam Wooridul Spine Hospital; Wiltse Memorial Hospital; Seoul National University Bundang Hospital; Kangnam Sacred Heart Hospital; and The Catholic University of Korea Daejeon St. Mary’s Hospital).

### Study group

The study sample will be 100 adults, 20–80 years of age, who present with radiating pain in the lower extremities due to a lumbar disc herniation. The method for sample size calculation is provided below. Fifty participants will be allocated each to the interlaminar full-endoscopic group and microscopic discectomy group. The equivalence between the two groups at baseline will be ascertained. Participants will be recruited from the five participating hospitals.

### Inclusion criteria

The inclusion criteria are as follows: patients for whom conservative treatment has failed and who are considered suitable for decompression surgery; age, 20–80 years; diagnosis of single-level lumbar disc herniation; radiating pain to the lower extremities, with a pain score > 4 on a 10-point visual analog scale (VAS); lumbar disc herniation cases wherein both interlaminar full-endoscopic discectomy and conventional microscopic discectomy are considered possible and appropriate by an operator; ability to follow instructions and to provide consent for participation; and willingness to comply with the trial’s follow-up protocol. The following types of disc herniation will be included: protruded, extruded, and migrated types of disc herniation in the central canal and subarticular zones [26].

### Exclusion criteria

The exclusion criteria are as follows: the presence of a spondylolisthesis (Meyerding grade ≥ II); spinal stenosis of more than a moderate degree (Schizas classification grade ≥ B) [27]; history of lumbar spinal surgery at the same level, including recurrent disc herniation; the presence of degenerative lumbar scoliosis (Cobb angle > 20°); other spinal diseases (e.g., ankylosing spondylitis, spine tumor, fracture, or neurologic disorders); and “any other” patient characteristic or disorders that the surgeons consider inappropriate for participation, including extreme sensitivity to pain, myofascial pain syndrome, history of paresis, and severe knee joint osteoarthritis. Patients with a sequestrated disc herniation and those with foraminal and extraforaminal disc herniation will be excluded [26].

### Recruitment

This will be a multicenter RCT and will include patients who decide to proceed with a one-level discectomy for lumbar disc herniation at each of the five participating hospitals between June 2021 and December 2024. There will be no recruitment via social media. The study researchers from each of the five participating hospitals will screen potential participants to determine their eligibility and willingness to participate.

### Data collection

After providing informed consent, participants will be enrolled in the study and will undergo baseline assessments, including the following: magnetic resonance images (MRIs), simple radiographs, the Oswestry Disability Index (ODI) score, the EuroQol-5-dimension-5-level (EQ-5D-5L) questionnaire, and the 10-point pain VAS scale, and demographics and baseline patient characteristics [including age, gender, occupation, comorbidity (diabetes mellitus, hypertension), bone mineral density, body mass index, medical/surgical history, smoking/drinking habits, physical examination, and laboratory test]. The assessor will be blinded to participants’ personal information.

### Randomization and follow-up

After completing the baseline assessments, participants will be block-randomized into either the control (microscopy) or intervention (endoscopy) group, using a 1:1 allocation ratio, with a block size of four. The randomization list will be computer-generated and integrated into a web-based electronic case report form (eCRF) platform (iCReaT; internet-based clinical research and trial, icreat.nih.go.kr). The central randomization will be conducted by a contract research organization company (Helptrial), and the data will be accessible only to the trial’s authorized researchers. The randomization of patients to either the control or intervention group will be presented to the study surgeons in each participating hospital using consecutively numbered opaque envelopes.

**Fig. 1:**
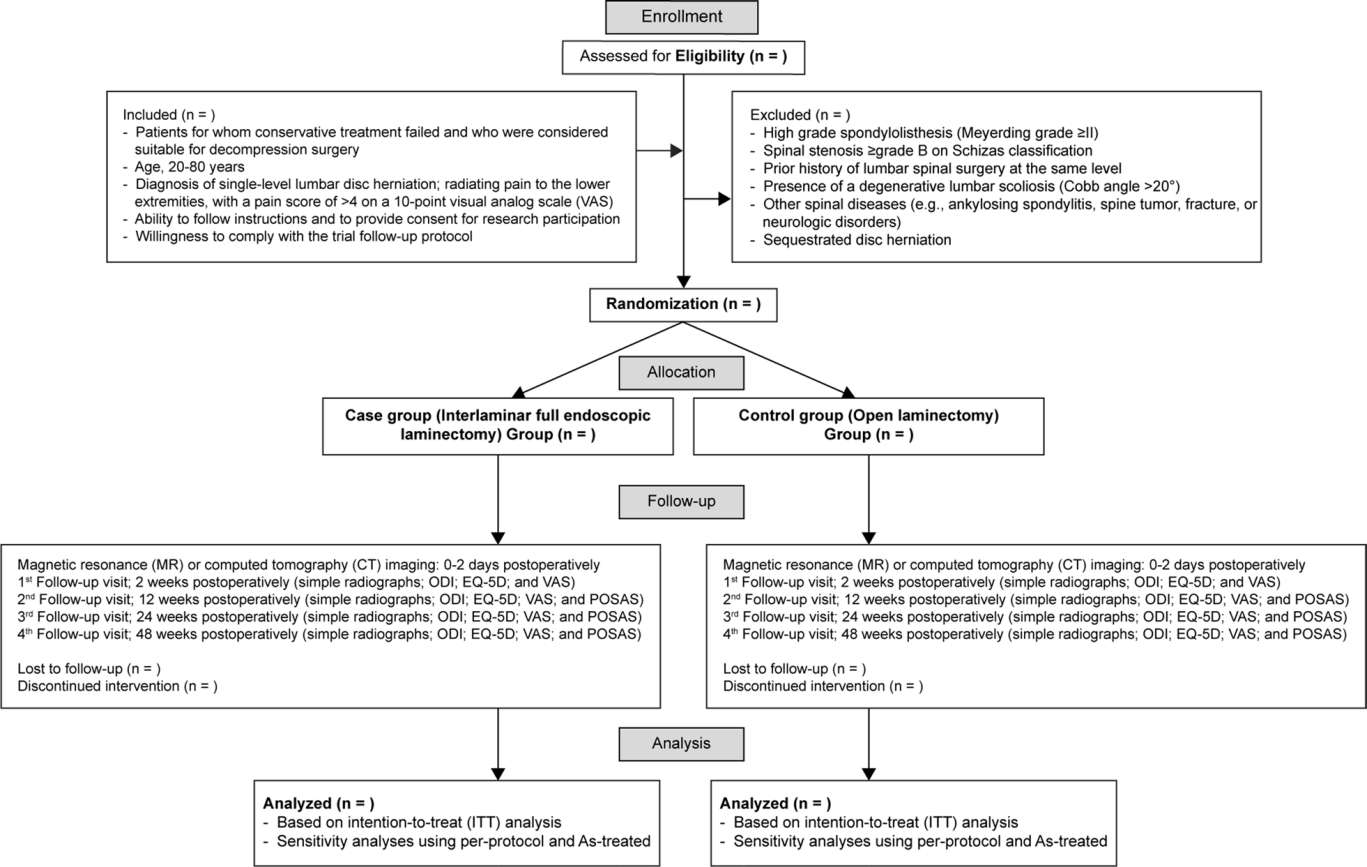
CONSORT study flow diagram for the trial protocol

To evaluate the primary and secondary outcomes, follow-up assessments will be planned for each participant at 2 weeks and at 3, 6, and 12 months after surgery. An independent researcher will perform the assessments at each time-point of follow-up. Phone interviews will be used under unavoidable circumstances where in-person follow-up is not possible (Fig. 1).

### Blinding

All the primary and secondary outcomes will be assessed at each participating hospital by a single assessor, who will be blinded to group allocation. The patients themselves who are assessors of the patient-reported outcomes (the VAS score of back and leg pain, EQ-5D-5L score, and ODI score) are blinded (i.e., they are not informed of their assignment). The investigators of the patient-reported outcomes are also blinded.

Because blinding of patients is an integral part of this study, the research personnel and healthcare providers will not disclose surgical information to patients throughout the study. However, blinding may fail due to other available information such as operative time, size of the skin incision, and hospital cost. Therefore, patients will be asked to guess the surgery they underwent, and the extent of successful blinding will be assessed based on this parameter.

Postoperative surgical scarring, the extent of disc removed, and the presence/absence of injury to the facet joint on postoperative MRI or computed tomography (CT) images will be measured by a blinded assessor. Complications observed on simple radiographs, adverse events and the severity of these events, as well as surgery-related events, will be evaluated and managed by a blinded assessor. Surgeons will be made aware of the procedure performed (interlaminar full-endoscopic discectomy or microscopic discectomy) in each case. This information will not be revealed by either the participants or surgeons to the assessor. If unblinding is required, the assessor will be required to submit a justification to the trial team based on the assessment findings.

### Surgical interventions

#### Active intervention: interlaminar full-endoscopic discectomy

The technical procedure for interlaminar full-endoscopic discectomy [28], including recent updates, is well described in the literature [3, 4, 29–33]. The spine surgeons are familiar with this approach due to its similarity to the conventional posterior approach, except for creating a working channel for the endoscope and spinal instrument [3, 4, 32]. The procedure is performed under general or spinal anesthesia with the participant in the prone. The surgical table is bent approximately at the level of the lower lumbar spine, with appropriate flexion of the hip and knees; this position widens the interlaminar window [3, 4]. A 5–7-mm skin incision is created at the entry point for the working channel, approximately 1 cm from the midline, at the level of the symptomatic herniated disc, visualized by intraoperative C-arm anteroposterior fluoroscopy imaging, as previously described [30]. The working channel is inserted and positioned at the target point over the ligamentum flavum with sufficient subdermal fascia dissection. After optimal positioning of the working channel at the targeted location, the endoscope is inserted into the working channel under sufficient irrigation along with saline solution [3, 4, 30, 32, 33].

**Fig. 2:**
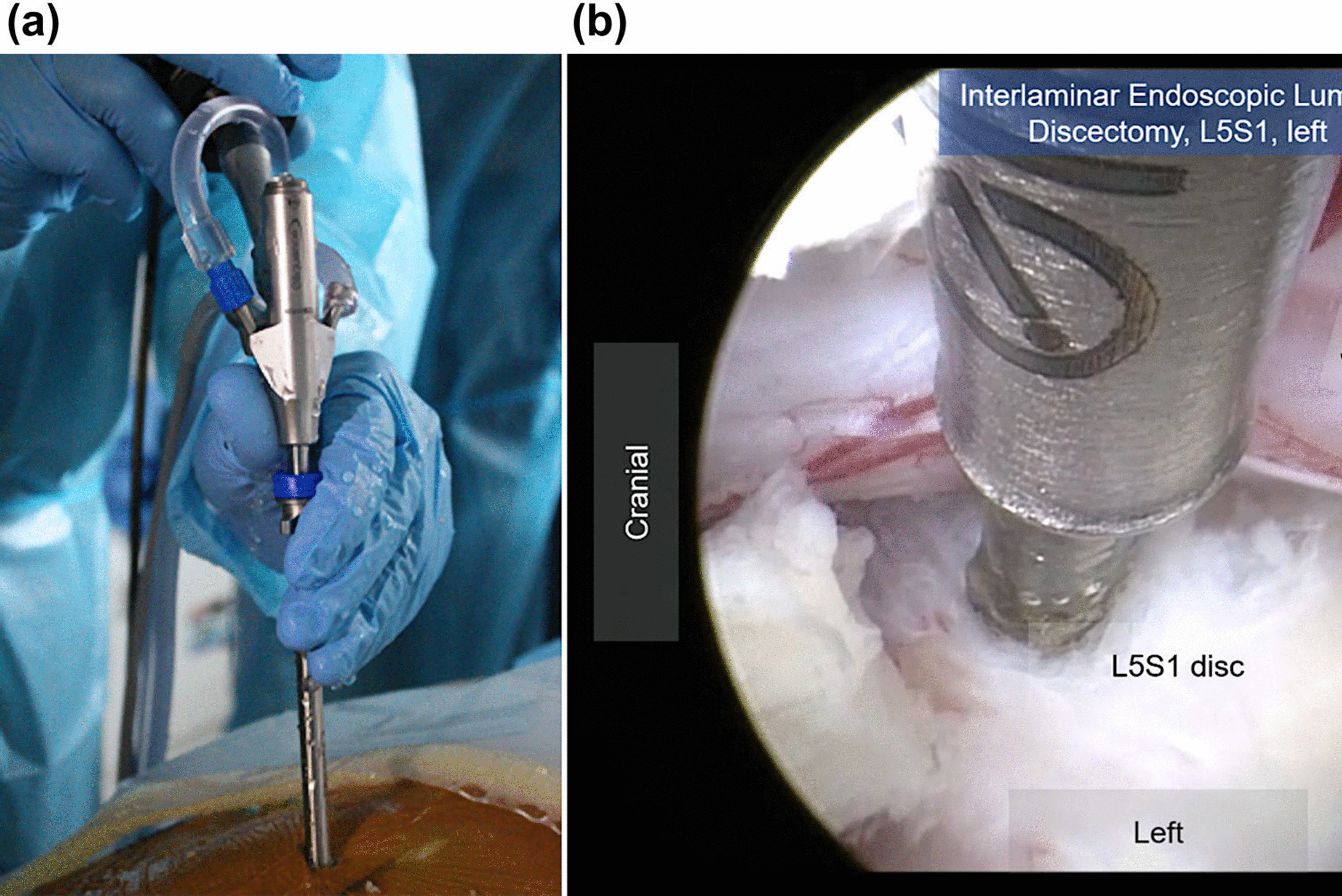
**a** Operative field of interlaminar full-endoscopic discectomy. **b** Intraoperative endoscopic view, showing the disc space and decompressed left S1 nerve root

Surgery is performed by inserting the required spinal surgical instruments (bipolar radiofrequency cauterization devices, burrs, Kerrison punches, and pituitary rongeurs) through the working channel. The paravertebral muscle is coagulated to identify the border of the interlaminar window [3, 4, 30]. The ligamentum flavum is either resected using a punch or split using a probe at the level of the tip of the descending facet [3, 11, 30, 31]. The discectomy is performed in a fully endoscopic, minimally invasive manner (Fig. 2).

#### Control intervention: conventional microscopic discectomy

For microscopic discectomy, the target lumbar level of symptomatic disc herniation is again visualized under C-arm intraoperative fluoroscopy, and a 2.5-cm midline incision is made over the target level. The paraspinal muscle is detached from the spinous process and lamina, and detached muscle towing is performed in a minimally invasive fashion through the small skin incision under microscopic visualization. Minimal laminotomy is then performed using a burr, and Kerrison punches under microscopic visualization. After the partial removal of the ligamentum flavum under the lamina, discal impingement of the spinal roots and dura is verified. The spinal nerve root is retracted using a root retractor, and the herniated disc is removed by pituitary forceps below the retracted nerve root. Following discectomy, the surgical field is verified for any remnant disc. This marks the end of the procedure.

### Measured outcomes

A complete description of the time-points at which the data on the primary and secondary outcomes will be collected is provided in Table 1.

**Table 1:** Evaluation schedule

Visit type	Screening	Surgical intervention	Follow-up			
Visit	1	2	3	4	5	6
Visit week	4–0 weeks	0–2 days	2 weeks	12 weeks	24 weeks	52 weeks
			± 5 days	± 4 weeks	± 8 weeks	± 8 weeks
Informed consent	■					
Demographics^*^	■					
Inclusion/Exclusion	■					
Randomization		■				
Surgery		■				
MRI (or CT)†	■	■				
Simple radiographs	■		■	■	■	■
ODI	■		■	■	■	■
EQ-5D-5L	■		■	■	■	■
VAS	■		■	■	■	■
POSAS				■	■	■
Other survey‡			■	■	■	■
Adverse events		■	■	■	■	■

POSAS, Patient and Observer Scar Assessment Scale

^*^Baseline patient characteristics, including age, gender, occupation, comorbidity (diabetes mellitus, hypertension), bone mineral density, body mass index, medical/surgical history, smoking/drinking habits, physical examination, and laboratory test

^†^CT imaging used when MRI cannot be performed

^‡^Including surgery satisfaction, walking time/distance, and return to daily activities after surgery

#### Primary outcome

The primary outcome will be the efficacy of the surgical intervention (interlaminar full-endoscopic discectomy or conventional microscopic discectomy), measured using the Oswestry Disability Index (ODI) score at 12 months [34, 35]. The ODI is the most valuable tool for evaluating patient-reported functional outcomes for lumbar spinal disabilities in a clinical setting [34, 35]**.** The ODI evaluates the level of function on activities of daily living for patients with low back pain across the following 10 areas: pain intensity, personal care, lifting, walking, sitting, standing, sleeping, sex life, social life, and traveling. Each section of the ODI is scored on a 5-point scale, with a score of 5 representing the most severe disability. The total ODI score is used for analysis, calculated as the sum of the scores across the 10 areas divided by the total possible score, and expressed as a percentage (i.e., multiplied by 100). For all unanswered questions, the total possible score is reduced by five. The highest score is recorded if the participant checks more than one answer. The ODI will be administered and scored by the assessor, which will be recorded in the eCRF system.

#### Secondary outcomes

The following secondary outcomes will be included in the analysis: patient-reported outcomes, clinical outcomes, radiographic outcomes, and adverse events. Patient-reported outcomes are as follows: (1) presence and severity of low back pain and pain radiating to the lower-extremities, measured using a 10-point VAS score, ranging from “0” (no pain) to “10” (severe pain); (2) quality of life (QOL), measured using the EuroQol-5-dimension-5-level (EQ-5D-5L) questionnaire, which consists of five questions, with the total score ranging between “0” and “1,” with a higher score indicating a better QOL [36]; (3) satisfaction with the surgery; and (4) walking distance/time and time to return to daily activities after surgery. The following clinical outcomes will be measured: (1) postoperative surgical scarring, measured using the Patient and Observer Scar Assessment Scale (POSAS; version 2.0), which consists of six items scored on a 10-point system, with a score of “6” (i.e., a score of ‘1’ on each item) indicative of normal skin and a score of “60” (i.e., a score of “10” on each item) indicative of the “worst scar imaginable” and (2) surgery-related variables, namely postoperative drainage (mL), operative time (min), duration of hospitalization (h), and postoperative creatine kinase. The following radiographic outcomes will be obtained: (1) the extent of disc removed and injury to the facet joint, measured using postoperative MR or CT images, and (2) simple radiographs will be used for measuring other complications during the follow-up period. We will use the Carragee classification to evaluate MRI outcomes [37]. The dimensions of the disc on an axial image with the greatest encroachment will be directly measured [37]. The area of the herniated disc encroaching into the canal space (disc area) and hemiarea of the herniated disc (hemidisc area) will be calculated by using the following linear measurements on pre- and postoperative MRIs: 1) the longest anterior–posterior disc length and 2) width of the herniated disc at the midpoint of its posterior protrusion (mid anteroposterior disc width) [37]. To evaluate facet joint injury, the width of the medial facet on an axial image with the greatest width of the facet joint will be measured on pre- and postoperative MRIs [37]. Radiographs will be obtained in the anteroposterior, lateral, flexion, and extension views, and spondylolisthesis and segmental instability at the target surgical level will be scored based on these images. Radiographs will also be obtained at baseline and during each follow-up session. Preoperative spinal MRI will be systematically conducted in the sagittal and axial planes to determine the type and location of the disc herniation. Safety will be evaluated based on the number of adverse events and the severity of these events, as well as surgery-related events. Adverse events, including postoperative infection and recurrence of disc herniation, will be recorded at each follow-up session. Recurrence of disc herniation will be diagnosed on MRI when newly developed radiculopathy is suspected during the follow-up period. Adverse events will be reported to the surgeon by the participant or the assessor and will be recorded in the electronic database. The data on patient-reported outcomes, clinical outcomes, plain radiographs, and adverse events will be collected at baseline and at each follow-up session (2 weeks and 3, 6, and 12 months after surgery). The assessor will manage and evaluate outcomes and record them in the eCRF system (Table 1).

### Statistical analyses

All statistical analyses will be performed using SAS Enterprise Guide 4 (SAS Institute Inc., Cary, North Carolina, USA). Before performing the main comparative statistical analyses, we will assess whether the baseline variables of the two groups are visually balanced.

An intention-to-treat strategy will be implemented in this non-inferiority RCT. In addition, sensitivity analyses using per-protocol and As-treated will be conducted to identify the deviations from the original assignments during randomization. Participants excluded before or after surgery will not be considered for analysis or replaced, thus avoiding the risk of bias in allocation concealment.

The primary outcome (the ODI score at 12 months post-surgery) will be compared between the two groups. Interlaminar full-endoscopic discectomy will be considered equivalent to microscopic discectomy concerning surgical outcomes if the 95% confidence interval (CI) of the treatment difference value of the Interlaminar full-endoscopic discectomy group is included in the equivalence limit of 12.8 points.

To analyze the time-dependent change in secondary patient-reported and clinical outcomes (i.e., VAS pain scores for the back and lower extremities and ODI, EQ-5D, and POSAS scores), a linear mixed model repeated-measures analysis of variance will be used. Time will be regarded as a categorical variable (at 2 weeks and at 3, 6, and 12 months) and analyzed to evaluate serial changes from baseline, within each group, and between the two groups at each session, with a post hoc test used for any significant time- and group-differences identified.

Chi-square test for categorical variables and Student’s t-test for continuous variables will be used to analyze other clinical and radiographic outcomes and adverse effects between the two groups. The collected data’s distribution will be evaluated using the Shapiro–Wilk test, with a two-sided P-value. Normally distributed continuous variables will be reported as means and standard deviations (SDs), with non-normally distributed continuous variables reported as medians and interquartile ranges. Categorical variables will be reported as count and percentage (%).

### Data management

Participant data will be anonymized and entered into the iCReaT platform created by the Korean government to allow researchers and investigators to input the research data safely and directly. The iCReaT platform is equipped with a web-based encryption system to protect the research data from unauthorized access and disclosure, and it will be accessible only to the principal investigator and designated statistical analysts. The e-CRF system will be used for this clinical trial. The iCReaT will be managed by specialized clinical research coordinators in each hospital and via a contract with a specialized company with extensive experience in eCRF management. For clinical trial monitoring, both on-site and in-house monitoring, using the electronic data capture system will be conducted by designated monitoring researchers.

### Sample size justification

In this trial, 100 participants will be recruited, with 50 in each group. Based on previous studies by Copay et al., the non-inferiority margin is 12.8 points and the maximal clinically accepted ODI difference is 12.8 points, with an SD of 17.1 points at 1-year after endoscopic discectomy [21, 35]. Based on the non-inferiority margin of 12.8, 50 participants will be required in each group, with an alpha value of 0.05, a power of 0.90, a one-sided 95% CI, and a loss to follow-up of 20%. Power Analysis and Sample Size software (version 15; NCSS, Kaysville, UT, USA) was used to calculate the sample size.

## Discussion

Previous studies have reported that interlaminar full-endoscopic discectomy offers similar clinical outcomes but is less invasive compared to open microscopic discectomy [18–22]. However, evidence supporting the efficacy and safety of interlaminar full-endoscopic discectomy is limited by the type of research design previously applied, i.e., retrospective, single-center prospective designs [7, 18–25].

This trial will be the most valuable, multicenter, prospective RCT to evaluate and to comparatively analyze the efficacy, safety, and applicability of interlaminar full-endoscopic discectomy, compared with those of open discectomy, in patients with lumbar disc herniation. The quality of the evidence will be improved by adequate sample size, blinded assessments, and prospective registration from multiple centers to reduce bias. This will ensure that the two approaches are evaluated equivalently. We anticipate that this high-quality evidence will provide a clear conclusion on the efficacy and safety of interlaminar full-endoscopic discectomy as an alternative option, with the same surgical outcome and less invasiveness, for the treatment of lumbar disc herniation.

## Data Availability

The electronic database server (iCReaT) will not be publicly accessible. Access to the data set is provided only to the Data Management Committee of the Korean Government Research Consortium. The study findings will be published in a peer-reviewed journal.

## Abbreviations

CI: Confidence interval
CT: Computed tomography
eCRF: Electronic case report file
EQ-5D-5L: EuroQol 5-dimension 5 level
iCReaT: Internet-based clinical research and trial
MR: Magnetic resonance
ODI: Oswestry Disability Index
POSAS: Patient and Observer Scar Assessment Scale
QOL: Quality of life
RCT: Randomized controlled trial
SD: Standard deviation
VAS: Visual analog scale
